# Aqueous Solution Chemistry of Ammonium Cation in the Auger Time Window

**DOI:** 10.1038/s41598-017-00756-x

**Published:** 2017-04-07

**Authors:** Daniel Hollas, Marvin N. Pohl, Robert Seidel, Emad F. Aziz, Petr Slavíček, Bernd Winter

**Affiliations:** 1 Department of Physical Chemistry, University of Chemistry and Technology, Prague, Technická 5, 16628 Prague Czech Republic; 2 Helmholtz-Zentrum Berlin für Materialien und Energie, Methods for Material Development, Albert-Einstein-Straße 15, D-12489 Berlin, Germany; 3 School of Chemistry, Monash University, 3800 Clayton, Victoria, Australia; 4 Department of Physics, Freie Universität Berlin, Arnimallee 14, D-141595 Berlin, Germany; 5 J. Heyrovský Institute of Physical Chemistry, Dolejškova 3, 18223 Prague 8, Czech Republic; 6 Fritz-Haber-Institut der Max-Planck-Gesellschaft, Faradayweg 4-6, D-14195 Berlin, Germany

## Abstract

We report on chemical reactions triggered by core-level ionization of ammonium (${{\rm{NH}}}_{4}^{+}$) cation in aqueous solution. Based on a combination of photoemission experiments from a liquid microjet and high-level *ab initio* simulations, we identified simultaneous single and double proton transfer occurring on a very short timescale spanned by the Auger-decay lifetime. Molecular dynamics simulations indicate that the proton transfer to a neighboring water molecule leads to essentially complete formation of H_3_O^+^ (aq) and core-ionized ammonia ${({{\rm{NH}}}_{3}^{+})}^{\ast }$(aq) within the ~7 fs lifetime of the nitrogen 1s core hole. A second proton transfer leads to a transient structure with the proton shared between the remaining NH_2_ moiety and another water molecule in the hydration shell. These ultrafast proton transfers are stimulated by very strong hydrogen bonds between the ammonium cation and water. Experimentally, the proton transfer dynamics is identified from an emerging signal at the high-kinetic energy side of the Auger-electron spectrum in analogy to observations made for other hydrogen-bonded aqueous solutions. The present study represents the most pronounced charge separation observed upon core ionization in liquids so far.

## Introduction

Electron spectroscopy using high energetic X-ray radiation has become a thriving method for electronic-structure investigations of matter^1^. For example, X-ray-based spectroscopies contribute significantly to the ongoing discussion on water structure in liquid phase^2–4^. X-rays are also known to trigger various chemical reactions by core ionization which leads to the formation of highly excited radicals^5, 6^. Such processes play an important role, for instance, in the radiation damage of biomolecules^7^. The mechanistic details are not yet fully understood, mostly due to the ultrashort timescale of the elementary relaxation processes involving both electron and nuclear motion^8^, which are difficult to access by experiment. In addition, X-ray studies from aqueous phase, particularly those based on electron detection, have been challenging because of the short electron mean free path. The introduction of the liquid-microjet technique has overcome this major problem and enabled liquid-phase photoelectron spectroscopy. Valuable information on the electronic structure, including valence and core-level binding energies of solute and solvent, has been obtained since then^9, 10^. In addition, spectroscopy of electrons generated by second-order (relaxation) processes has considerably contributed to our understanding of the ultrafast electron and nuclear dynamics initiated by X-rays^11, 12^.

Ionization of molecules or ions by high-energy radiation leads to the formation of excited species which relax either via radiative (X-ray fluorescence) or non-radiative (Auger-type autoionization) decay channels. In the case of autoionization, the core hole formed upon core ionization is refilled by a valence electron while another valence electron is ejected. Non-radiative decay is dominant for light atoms. It can be a local (Auger decay) or non-local process with different autoionization mechanisms^13^. Non-local processes have been recently explored for hydrogen-bonded small molecules in aqueous solution, including water (aq)^5, 14^, hydrogen peroxide (aq)^15^, ammonia (aq)^16^, glycine (aq)^16^, formaldehyde (aq)^17^, formaldimine (aq)^17^, and hydrogen sulfide (aq)^17^. For a given molecule AH^*q*^ (aq) with charge *q* in aqueous phase, core ionization leads to the formation of highly excited radicals ${({{\rm{AH}}}^{q+1})}^{\ast }$. The asterisk denotes a core-hole excited state. In an Auger process, this singly ionized state autoionizes locally by forming a doubly-ionized species AH^*q*+2^. We denote the respective local two-hole final state as *2* *h*. This notation is used to distinguish from final states produced by non-local autoionization processes of ${({{\rm{AH}}}^{q+1})}^{\ast }$ where electronic relaxation includes neighboring molecules. The core hole is then refilled by a valence electron, but instead of ejecting a local Auger electron, an electron is emitted from a water molecule in the first hydration shell. Accordingly, this so-called intermolecular Coulombic decay (ICD) creates two positive charges shared between two molecular partners, e.g. ${{\rm{AH}}}^{q+1}\cdots {{\rm{H}}}_{2}{{\rm{O}}}^{+}$. This delocalized final state is referred to as 1*h*1*h* (one-hole-one-hole). The above mentioned ionization and relaxation processes can be expressed as:

1 $$\begin{array}{cc}{\rm{Core}}\,\mathrm{ionization}: & h\nu +{{\rm{AH}}}^{q}\cdots {{\rm{H}}}_{2}{\rm{O}}\to {({{\rm{AH}}}^{q+1})}^{\ast }\cdots {{\rm{H}}}_{2}{\rm{O}}+{{\rm{e}}}_{{\rm{photo}}},\end{array}$$

2 $$\begin{array}{cc}{\rm{Auger}}\,\mathrm{decay}: & {({{\rm{AH}}}^{q+1})}^{\ast }\cdots {{\rm{H}}}_{2}{\rm{O}}\to {{\rm{AH}}}^{q+2}\cdots {{\rm{H}}}_{2}{\rm{O}}+{{\rm{e}}}_{{\rm{Auger}}}\end{array},$$

3 $$\begin{array}{cc}\mathrm{ICD}: & {({{\rm{AH}}}^{q+1})}^{\ast }\cdots {{\rm{H}}}_{2}{\rm{O}}\to {{\rm{AH}}}^{q+1}\cdots {{\rm{H}}}_{2}{{\rm{O}}}^{+}+{{\rm{e}}}_{{\rm{ICD}}}\end{array}.$$

Other types of non-local relaxation processes involving even more molecular units such as the electron-transfer mediated decay (ETMD) are also possible^13, 18^. The non-local decay processes can have a considerable spectral contribution, sometimes comparable to that of the local Auger process. This is the case when autoionization is accompanied by proton dynamics^5, 12^. In the so-called proton-transfer mediated charge separation (PTM-CS) process^5^, the core-ionized molecule releases a proton which is then shared with another water molecule from the hydration shell, forming a transient structure analogous to the Zundel cation in water, in which the proton is shared equally between the two species^19^:

4 $${({{\rm{AH}}}^{q+1})}^{\ast }\cdots {{\rm{H}}}_{2}{\rm{O}}\to [{{\rm{A}}}^{q\ast }\cdots {{\rm{H}}}^{+}\cdots {{\rm{H}}}_{2}{\rm{O}}].$$

Note that unlike Equations (2) and (3), Equation (4) does not consider the final autoionization event. The electron can be ejected either from the molecule A (local) or the neighboring water unit (non-local); the final products are $[{{\rm{A}}}^{q+1}\cdots {{\rm{H}}}^{+}\cdots {{\rm{H}}}_{2}{\rm{O}}]$ or $[{{\rm{A}}}^{q}\cdots {{\rm{H}}}^{+}\cdots {{\rm{H}}}_{2}{{\rm{O}}}^{+}]$, respectively. The charge separation, leading to the transient core-excited species, is thus supported by the proton motion which has been identified in previous studies^20–25^. Theoretical analysis of this process in liquid water has shown that Zundel-type transients have an increased probability to decay via ICD^14^, creating 1*h*1*h* states. PTM-CS is experimentally identified by an isotope effect in the autoionization spectra. Specifically, the *1* *h1* *h* states have a lower energy than the *2* *h* states (by approximately 5 eV in liquid water^5^) due to the reduced Coulomb repulsion, giving rise to increased signal intensity at the high-kinetic energy side of the respective Auger spectra. Notice that local Auger decay for the manifold of the Zundel-analogue structures will also produce *1* *h1* *h* states, with energies similar to the ones produced by ICD^12, 14^. Experimentally, the occurrence of PTM-CS is identified from a larger intensity of the characteristic *1* *h1* *h* signal for the lighter isotope, i.e. H_2_O in normal liquid water versus D_2_O in deuterated liquid water. This is because the lighter and faster moving proton forms the Zundel-type structures more efficiently compared to the heavier deuteron within the core-hole lifetime (approximately 4 fs for O 1s^25^ and 6.4 fs for N 1s^26^).

It has been found from studies of other hydrogen-bonded solutes in aqueous solutions that the probability of PTM-CS strongly correlates with hydrogen-bond strength, which naturally makes this particular spectral fingerprint a rather sensitive probe of hydration structure^12^. This dependence made us to explore how PTM-CS manifests in a much stronger hydrogen-bonded system as compared to the ones studied so far. A large effect is expected when going from neutral to cationic molecule. Our study case is ammonium in water, ${{\rm{NH}}}_{4}^{+}\,$(aq), which forms stronger hydrogen bonds with water than does neutral NH_3_^27^. The ${{\rm{NH}}}_{4}^{+}\cdots {{\rm{H}}}_{2}{\rm{O}}$ system is stabilized dominantly by a strong ion – dipole interaction. By theoretically analyzing the core-ionization-induced relaxation processes of ${{\rm{NH}}}_{4}^{+}$ (aq), we are able to predict how likely PTM-CS is and how an extra charge influences the structure of the transient species. Regarding the previously studied neutral molecules (H_2_O, H_2_O_2_, and NH_3_)^5, 15, 16^, our computational analysis addresses several aspects unique to ${{\rm{NH}}}_{4}^{+}$. First, having one more hydrogen atom than NH_3_, the probability for a proton transfer is expected to increase. Second, unlike NH_3_, ${{\rm{NH}}}_{4}^{+}$ is positively charged already before the core ionization resulting in a double positive charge after ionization. This increases both the Coulomb repulsion between the parent molecule and a proton, as well as the attraction between a water oxygen and a proton. Third, the ionized doubly-charged state possibly enables double-proton transfer. All these conditions are in favor of the PTM-CS process and should result in a much stronger isotope effect and possibly in the occurrence of additional spectral features than observed in all previous studies. Understanding the relaxation processes in ${{\rm{NH}}}_{4}^{+}$ (aq) is a prerequisite for analyzing autoionization spectra of several biologically relevant molecules, for instance amino acids in their different protonation states in aqueous phase.

## Methods

### Calculations

We have addressed three aspects in our calculations: (i) the structure of the ${{\rm{NH}}}_{4}^{+}$ ion in aqueous solution, (ii) the energetics of the autoionization processes, and (iii) the proton dynamics on the core-ionized potential energy surface (PES). The first aspect was approached by *ab initio* molecular dynamics (MD) simulations for the solution in thermal equilibrium. The energetics of the autoionization process was investigated using quantum chemical methods. The dynamics on the core-ionized PES were investigated using semi-classical *ab initio* MD simulations for finite-size cluster models.

The *ab initio* MD simulations of the solvated ammonium ion in thermal equilibrium were performed using the *QuickStep* module of the *CP2K* program^28, 29^, utilizing the mixed plane-wave/Gaussian basis set approach with periodic boundary conditions^30^. We applied the BLYP functional with molecularly optimized DZVP-MOLOPT-SR basis set^31^ and Goedecker-Teter-Hutter pseudopotentials^32^. The cutoff frequency for the plane waves was set to 400 Ry. The system consisted of 63 water molecules and one solute molecule, and the density was set to 1 g/ml. After initial equilibration for ~2 ps, the simulation was performed in the NVT ensemble for 23 ps with 0.5 fs time steps, keeping fixed temperature of 300 K. To model the nuclear quantum effects important for the PTM-CS process, we have used the approximate quantum thermostat approach based on the generalized Langevin equation (GLE)^33, 34^. Unlike more rigorous techniques such as path integral MD, the quantum thermostat naturally provides also approximate quantum momentum distributions^33^ needed for subsequent semi-classical simulations. It was previously shown that the quantum thermostat technique (albeit in slightly different implementation) is a good approximation to Wigner distributions even for anharmonic systems^35^. A proper sampling of momenta is of critical importance especially for ultrafast processes where the dynamics is dominated by wave packet dispersion rather than the slope of the potential^14, 36^.

To calculate the absolute energy position of the leading local Auger peak, we first calculated the energy of the leading Auger peak in the gas phase as the difference between the core-ionized state and the ground state of the doubly-ionized system at the CCSD(T)/cc-pCVTZ level. The core-ionized state was calculated with the maximum overlap method (MOM)^37, 38^ applied to the CCSD(T)/cc-pCVTZ electronic structure level. Furthermore, a relativistic correction due to the removal of the N 1s electron was added as described in ref. 39. The correction amounts to 0.43 eV for an oxygen atom, and 0.23 eV for a nitrogen atom. A constant solvent shift was approximated via the implicit solvation C-PCM model^40, 41^. The non-equilibrium approach was used because of the ultrafast nature of the Auger decay. The initial polarization was computed for ground-state configuration, and only the electronic part of the polarization was allowed to relax in the subsequent calculations of core and doubly ionized final states. The solute cavity was constructed using Bondi radii^42, 43^, multiplied by a factor of 1.2. This approach was tested for liquid water and aqueous ammonia where validation with experimental data is possible.

To investigate proton-transfer dynamics, we constructed a two-dimensional PES scan along the proton-transfer coordinate for the core-ionized ${({{\rm{NH}}}_{4}^{2+})}^{\ast }{({{\rm{H}}}_{2}{\rm{O}})}_{3}$ cluster using the MOM-MP2/cc-pCVDZ approach. The optimized ground-state geometry of the cluster was calculated at the MP2/cc-pCVTZ level with a counterpoise correction^44^. The static PES scan, however, does not provide conclusive information on whether the process of interest actually takes place. Therefore, we performed dynamical simulations of larger ${({{\rm{NH}}}_{4}^{2+})}^{\ast }{({{\rm{H}}}_{2}{\rm{O}})}_{20}$ clusters on the core-ionized PES calculated on-the-fly at the MOM-B3LYP/cc-pVDZ level. Initial geometries and velocities were taken from the *ab initio* CP2K simulation described above. The system was simulated for 10 fs, corresponding to the nitrogen core-hole lifetime (~6.4 fs^26^), and the time step was set to 0.25 fs. A total of 400 trajectories was launched. We repeated all simulations for the deuterated systems to model the isotope effect. We have carefully examined the convergence of the results with respect to the size of the cluster and the level of electronic structure theory; the results of this analysis can be found in the Supplementary Material. To speed up the calculations for larger clusters, we have implemented the MOM method in the development version of the GPU code TeraChem^45, 46^. We have validated our implementation against the results from the MOM method as implemented in the Q-Chem package^47^.

The geometric structure of the ${{\rm{NH}}}_{4}^{+}{({{\rm{H}}}_{2}{\rm{O}})}_{3}$ was optimized using *Gaussian* code^48^. The *ab initio* calculations using MOM-MP2 and MOM-CCSD(T) were done with the *QCHEM* program^47^ while MOM-B3LYP calculations were done using the development version of *TeraChem* code^45, 46^. All MD simulations were performed with the in-house *Abin* code^49^ while forces and energies were taken each timestep from an external *ab initio* program (either *QCHEM*, *CP2K* or *TeraChem*). The estimate of the interaction energies between hydrogen bonded dimers was done at the CCSD(T)/CBS level using the *MOLPRO* code^50^.

### Experiment

Photoelectron- and Auger-electron spectroscopy measurements were conducted at the U49/2-PGM-1 undulator beamline at the BESSY II synchrotron-radiation facility in Berlin. Auger-electron spectra associated with the nitrogen 1s ionization of aqueous ${{\rm{NH}}}_{4}^{+}$ were collected using 500 eV photon energy, illuminating a 25 μm diameter liquid microjet at a temperature of approximately 18 °C and traveling with a velocity of approximately 80 ms^−1^. Experimental details of the liquid-microjet technique have been described previously^51, 52^. Emitted electrons were detected using a hemispherical electron-energy analyzer at normal angle with respect to the polarization direction of the incident light. Since the angular distribution of second-order electrons is isotropic, the detection geometry has no effect on the data discussed here. The energy resolution of the U49 beamline was better than 230 meV at the photon energies used here and the energy resolution of the hemispherical energy analyzer was constant with kinetic energy, approximately 200 meV at 40 eV pass energy. Ammonium chloride aqueous solutions were prepared from NH_4_Cl salt (Sigma Aldrich # A9434, >99.5% purity) which was dissolved in neat liquid water, corresponding to 2 molar (M) ${{\rm{NH}}}_{4}^{+}$ concentration. The same procedure was applied for preparing a 2 M aqueous solutions of deuterated ammonium chloride, dissolving ND_4_Cl salt (Sigma Aldrich # 175676, >98% purity) in heavy water.

## Results and Discussion

In order to discuss the results of our combined theoretical and experimental studies on the proton-transfer mediated charge separation processes in aqueous ${{\rm{NH}}}_{4}^{+}$, we first present computation-based evidence for this process. We then show that the predicted behavior is in very good agreement with our experimental spectra.

**Figure 1:**
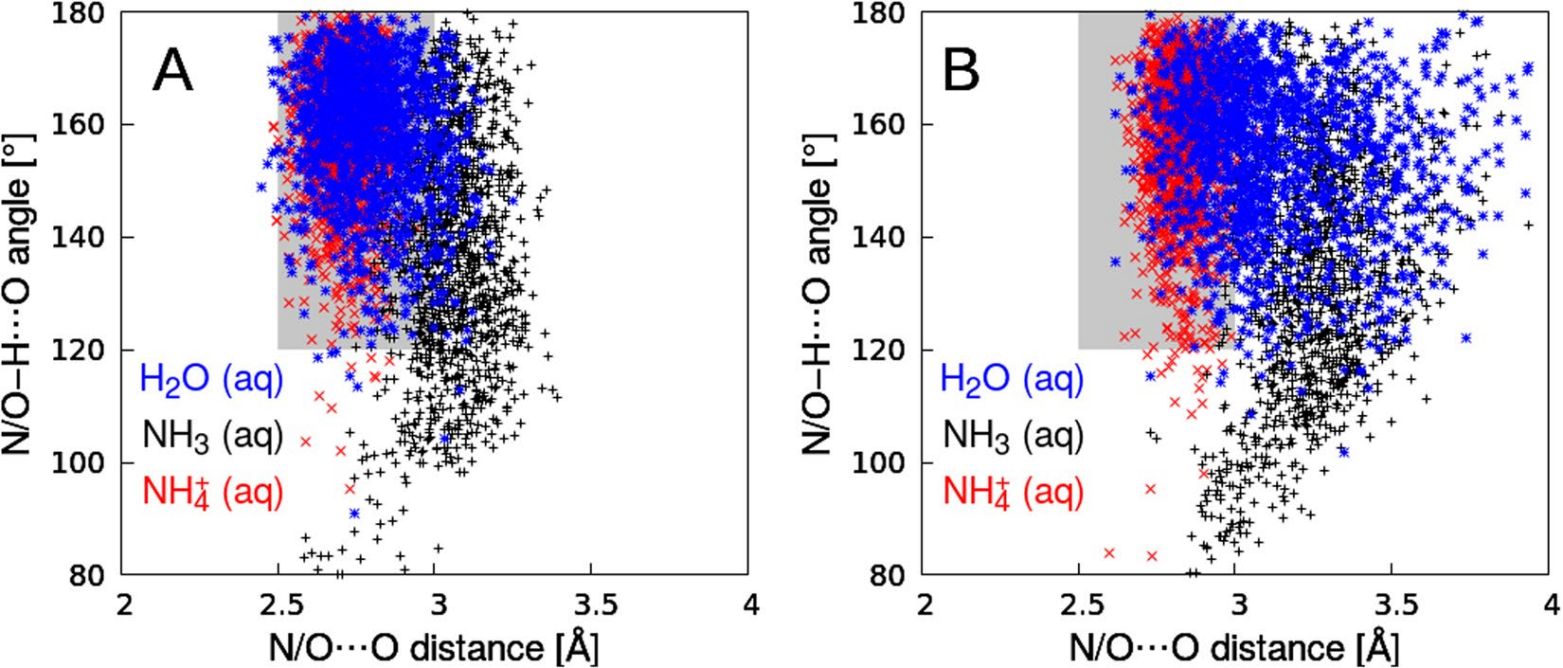
Hydrogen-bond strengths of liquid water (blue, square symbols), ammonia (black, plus symbols) and ammonium (red, cross symbols) aqueous solutions. Two parameters characterize the hydrogen-bond strength: The N$\cdots$O distance (O$\cdots$O for water) and the N/O–H$\cdots$O angle. Panel (A) shows data for the strongest hydrogen bond (i.e., shortest) and panel (B) corresponds to the second-strongest hydrogen bond. The shaded areas indicate the parameter ranges typically considered for strong hydrogen bonding^27^.

Let us start by analyzing the hydrogen-bond strength between water solvent and ${{\rm{NH}}}_{4}^{+}$ in the ground-state configuration. As we have shown before, PTM-CS gets more pronounced as the hydrogen bonding gets stronger^12^. While the ammonia molecule is a poor hydrogen-bond donor^16^, ${{\rm{NH}}}_{4}^{+}$ exhibits strong hydrogen bonding^27^. This can be inferred already from the analysis of molecular dimers. The ${{\rm{NH}}}_{4}^{+}\cdots {{\rm{H}}}_{2}{\rm{O}}$ complex is stabilized by 87 kJ/mol, which is much stronger compared to the ${{\rm{NH}}}_{3}\cdots {{\rm{H}}}_{2}{\rm{O}}$ (10 kJ/mol) and the ${{\rm{H}}}_{2}{\rm{O}}\cdots {{\rm{H}}}_{2}{\rm{O}}$ (21 kJ/mol) complexes, as calculated at the CCSD(T)/CBS level. We note that the NH_3_···H_2_O complex in which NH_3_ acts as a hydrogen-bond donor does not represent a true minimum on the potential energy surface. The values reported here were calculated for the geometry obtained via constrained minimization, which further highlights the weak hydrogen-bonding between neutral ammonia and water. The bond strengths correlate with the intermolecular distances between the heavy atoms (N/O and O) contributing to the hydrogen bonds in the dimers: ~2.67 Å bond length for ammonium, ~2.9 Å for water, and ~3.24 Å for ammonia. MD simulations of the fully hydrated solute in periodic boundary conditions provide a more detailed characterization of the hydrogen-bond strength. In Fig. 1 we show the proton densities (calculated in a quasi-classical way) projected onto two coordinates which characterize the strength of the hydrogen bonding: the O/N–O distance and the O/N–H···O angle. The optimum angle for a strong hydrogen bond is 180°, corresponding to perfect collinearity of the hydrogen bond. By taking water as a benchmark for a system with strong hydrogen bonds, we see from Fig. 1A that ${{\rm{NH}}}_{4}^{+}$ (aq) exhibits similar hydrogen bond lengths (2.5 Å–3.0 Å) and angles (120°–180°) for the strongest hydrogen bond. For NH_3_ on the other hand, both parameters (2.8 Å–3.3 Å and 100°–180°) are essentially outside the region of strong hydrogen bonding. Remarkably, even the second-strongest hydrogen bond in ${{\rm{NH}}}_{4}^{+}$ (aq) is almost as strong as the strongest hydrogen bond in H_2_O (aq), as presented in Fig. 1B. Note that ${{\rm{NH}}}_{4}^{+}$ (aq) can form up to four hydrogen donor-bonds to surrounding water molecules; the average coordination number is 3.3 according to our simulations. The existence of two strong hydrogen bonds in ${{\rm{NH}}}_{4}^{+}$ (aq) has crucial implications for the overall relaxation processes, potentially enabling the transfer of two protons upon core ionization as will be discussed next.

**Figure 2:**
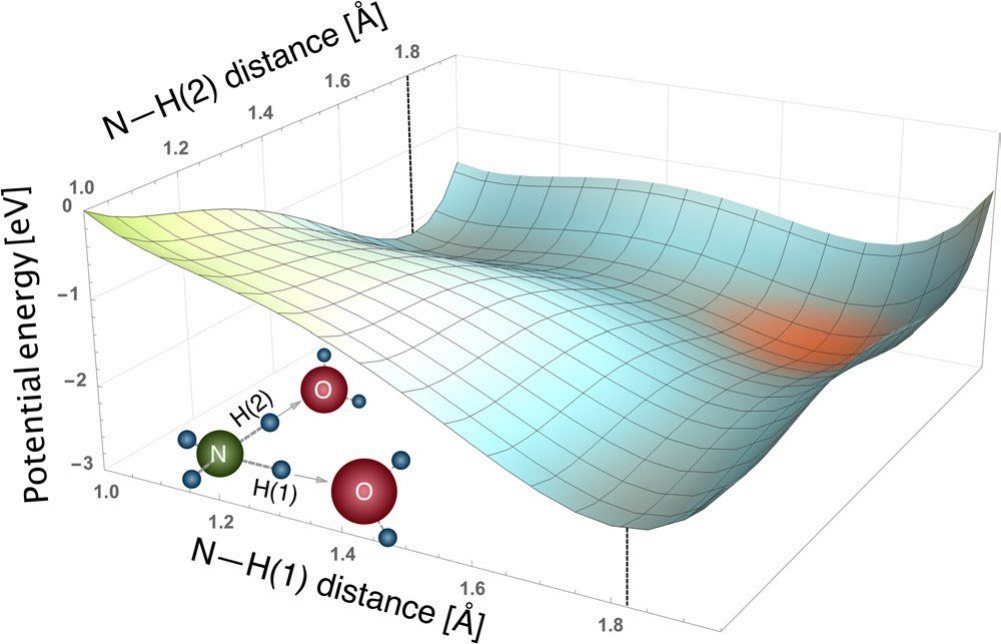
Unrelaxed two-dimensional cut through the potential-energy surface of a core-ionized ${{\rm{NH}}}_{4}^{+}{({{\rm{H}}}_{2}{\rm{O}})}_{3}$ cluster showing the electronic energy as a function of the N—H distances along the direction of two hydrogen bonds. The N—H ground state distance is 1.1 Å. The minimum energy corresponding to the fully transferred proton is at ~1.8 Å, marked by black dashed lines. Note that the third water molecule in the molecular sketch is omitted for clarity.

To explore the possibility of single and double proton transfer in ${{\rm{NH}}}_{4}^{+}$ (aq), we analyze the energetics of the proton transfer. Figure 2 shows the computed PES of micro-solvated ${{\rm{NH}}}_{4}^{+}{({{\rm{H}}}_{2}{\rm{O}})}_{3}$ for two protons which independently move from the nitrogen atom towards the oxygen atoms of adjacent water molecules. We chose three hydrating water molecules to mimic the average coordination number obtained from our MD simulations. The observed steep energy decrease along both proton-transfer coordinates implies that core-ionization-induced proton transfer is energetically favorable, even if two protons move simultaneously. Note that the minimum energy at ~1.8 Å N–H distance in Fig. 2 corresponds to the proton being fully transferred, forming H_3_O^+^ (aq). However, dynamical calculations are required to confirm that these processes actually occur during the ultrashort 6.4 fs nitrogen core-hole lifetime. The important question that arises is whether the complete proton-transfer reactions, as expressed in Equations (5) and (6) below, indeed occur, i.e. whether a new H^+^–O chemical bond forms before the autoionization event:

5 $$\begin{array}{cc}\mathrm{Single}{\rm{ \mbox{-} }}\mathrm{proton}\,\mathrm{transfer}: & {({{\rm{NH}}}_{4}^{2+})}^{\ast }\cdots {{\rm{H}}}_{2}{\rm{O}}\to {({{\rm{NH}}}_{3}^{+})}^{\ast }+{{\rm{H}}}_{3}{{\rm{O}}}^{+}\end{array},$$

6 $$\begin{array}{cc}\mathrm{Double}{\rm{ \mbox{-} }}\mathrm{proton}\,\mathrm{transfer}: & {({{\rm{NH}}}_{4}^{2+})}^{\ast }\cdots 2{{\rm{H}}}_{2}{\rm{O}}\to {({{\rm{NH}}}_{2})}^{\ast }+2{{\rm{H}}}_{3}{{\rm{O}}}^{+}.\end{array}$$

**Figure 3:**
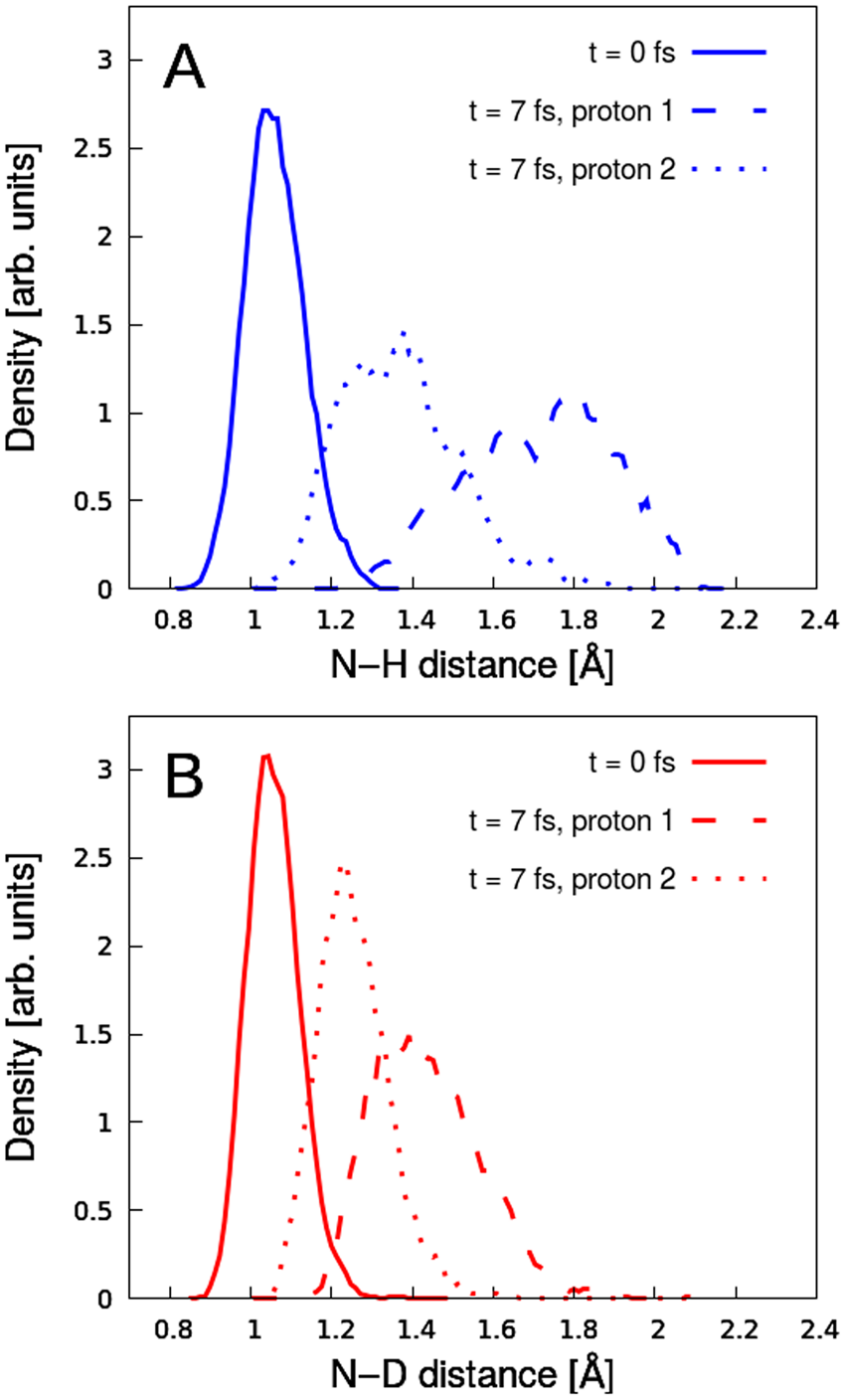
Time-dependent proton (deuteron) densities along the proton (deuteron) -transfer coordinates obtained from MD simulations on the core-ionized state of the (**A**) ${{\rm{NH}}}_{4}^{+}{({{\rm{H}}}_{{\rm{2}}}{\rm{O}})}_{20}$ and (**B**) ${{\rm{ND}}}_{4}^{+}{({{\rm{D}}}_{2}{\rm{O}})}_{20}$ clusters. The initial structures were taken from liquid-phase MD simulations of the solvated ammonium cation in the ground state. Densities along the strongest (dashed line) and second strongest (dotted line) N–H bonds are shown after 7 fs, together with the ground-state proton density (thick line).

To quantify how fast the proton transfer actually is, we have performed dynamical calculations on the N 1s core-ionized state for a larger number of hydration water molecules, ${{\rm{NH}}}_{4}^{2+}{({{\rm{H}}}_{2}{\rm{O}})}_{20}$ clusters. Figure 3 shows calculated proton densities projected onto the N–H/D coordinate for ${{\rm{NH}}}_{4}^{2+}{({{\rm{H}}}_{2}{\rm{O}})}_{20}$ clusters (Fig. 3A) and ${{\rm{ND}}}_{4}^{2+}{({{\rm{D}}}_{2}{\rm{O}})}_{20}$ clusters (Fig. 3B) at times *t* = 0 fs and *t* = 7 fs after core ionization. We observe that single-proton transfer in the case of ${{\rm{NH}}}_{4}^{+}$ (aq) is extremely fast, i.e. the process is practically completed within 7 fs. The center of the proton density curve for the strongest hydrogen bond is at ~1.8 Å (red curve in Fig. 3A), which is almost the minimum-energy distance according to Fig. 2. Note also that the proton density reaches as far as 2.1 Å. The other important observation from Fig. 3A is the considerable motion of the second strongest bonding proton, reaching a mean N–H distance of ~1.4 Å, which is half way toward its coordinated water oxygen. Although the dynamics is slowed down for the ${{\rm{ND}}}_{4}^{2+}{({{\rm{D}}}_{2}{\rm{O}})}_{20}$ cluster, it is still remarkably fast for the strongest bonding deuteron (red curve in Fig. 3B), comparable to the density distribution of the second proton motion for the ${{\rm{NH}}}_{4}^{2+}{({{\rm{H}}}_{2}{\rm{O}})}_{20}$ cluster. Note that MD simulations in previous studies revealed that the isotope effect in neutral NH_3_ (aq) is extremely small, almost unnoticeable^16^.

Experimentally, such or analogous reactions have never been observed within the <10 fs core-hole lifetime. As mentioned in the Introduction, PTM-CS will give rise to an electron signal at the high-kinetic energy side of the (local) N 1s Auger spectrum. It is difficult, though, to anticipate how the theoretically predicted double-proton transfer reflects in the measured spectra. In any case, we expect a large isotope effect in the autoionization spectra.

**Figure 4:**
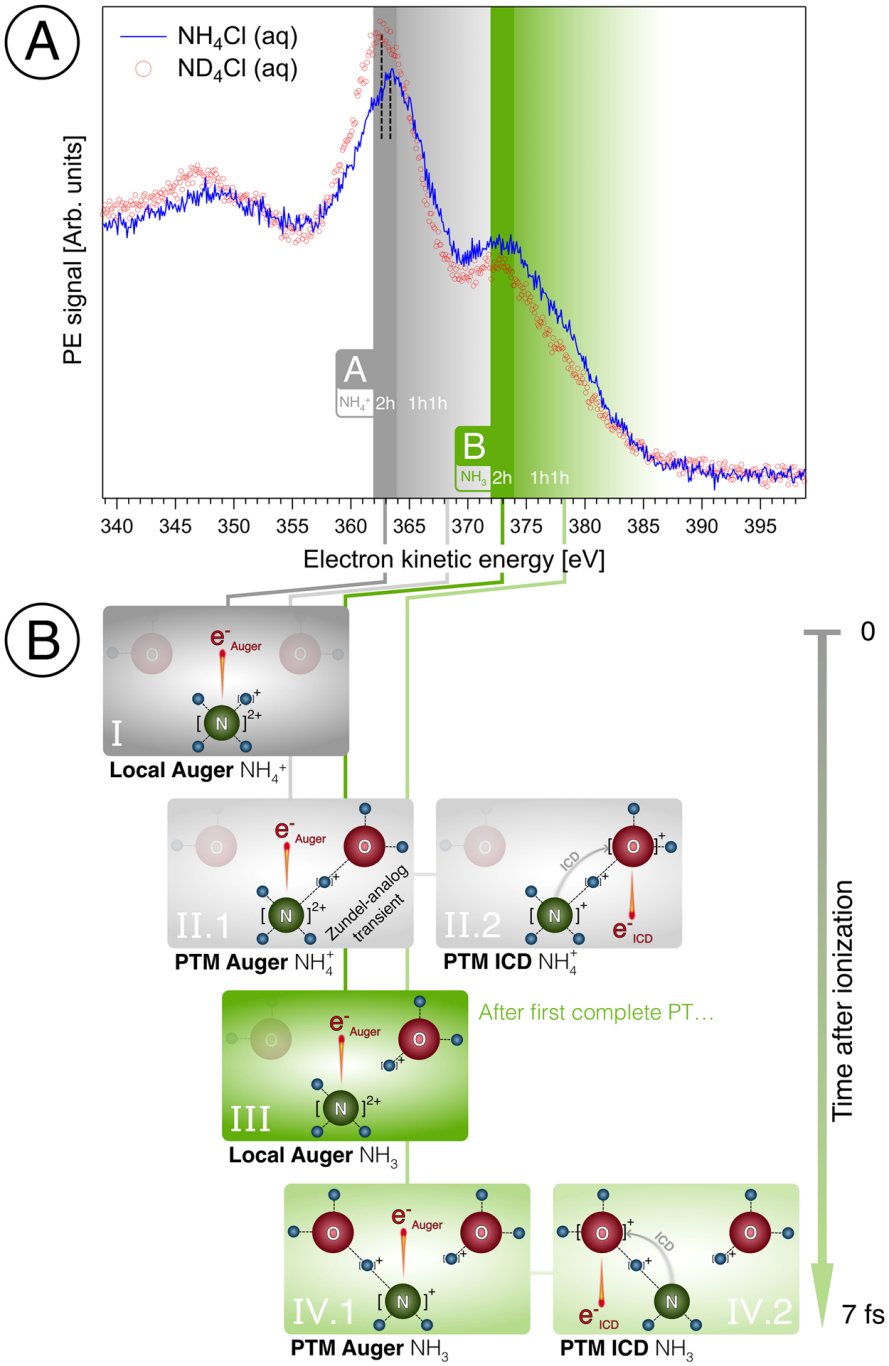
(**A**) Nitrogen 1s Auger/autoionization spectra from 2 M NH_4_Cl (blue curve) and 2 M ND_4_Cl (red circles) aqueous solution measured at 500 eV photon energy. Details as well as all labels in the figure are explained in the text. (**B**) Illustration of the double-proton transfer of ${{\rm{NH}}}_{4}^{+}$ in aqueous solution upon core ionization; only two water molecules are shown. (I) Auger process forming core-excited ${({{\rm{N}}{\rm{H}}}_{4}^{3+})}^{\ast }$ (aq) and Auger electron e_Auger_. (II) Auger decay and ICD of proton-transferred structure transients. (III) The complete transfer of the first proton from ${({{\rm{NH}}}_{4}^{2+})}^{\ast }$ (aq) to a neighbor water molecule forms ${{(\text{NH}}_{3}^{+})}^{\ast }$ (aq) + H_3_O^+^ (aq) within 7 fs. Subsequent local Auger decay is depicted. (IV) ${{(\text{NH}}_{3}^{+})}^{\ast }$ also releases a proton which travels only half way to another water molecule, forming a Zundel-analogue complex where the proton is shared between the remaining ${{\rm{NH}}}_{2}^{\ast }$ (aq) and a water molecule. Depiction of the subsequent autoionization processes of the transient structures by Auger decay (IV.1) and ICD and PTM-Auger (IV.2). These latter processes are the same as for NH_3_ aqueous solution. The vertical arrow indicates the temporal succession of processes I to IV, with IV being completed within 7 fs.

Auger/autoionization spectra from a 2 M NH_4_Cl (in H_2_O) and 2 M ND_4_Cl (in D_2_O) aqueous solution are shown in Fig. 4A. The photon energy was 500 eV which is well above the N 1s ionization energy (near 400 eV)^53^. Spectra are displayed as measured, but the intensities were scaled such that the areas under both curves are the same; this accounts for the assumption that differences in both photoionization and Auger decay cross sections for NH_4_Cl (aq) and ND_4_Cl (aq) are negligibly small. Two main observations can be made from Fig. 4A. First, both spectra exhibit a pronounced double-peak structure, with peaks centered at 362.5 eV KE (peak ***A***) and 373 eV KE (peak ***B***). An analogous spectral structure has not been observed in our previous studies on neutral solute molecules^5, 15, 16^. The second observation is that the intensities of both peaks exhibit a strong isotope effect which is in fact connected with single- and double-proton transfer as we will show below. Note that the isotope effect of peak ***A***, yielding larger electron-signal intensity at the high-kinetic energy side for the lighter isotope, correlates with an intensity loss on the lower-energy side near the peak maximum. A very similar isotope behavior has been observed for H_2_O (aq)^5, 14^, where signal at the high-energy side of the O 1s Auger spectrum is due to *1* *h1* *h* states produced from O 1s autoionization of proton-transferred transient structures, and the signal decrease at the low-energy side corresponds to the smaller abundance of *2* *h* states produced from local Auger decay^5^. We can therefore conclude that the peaks ***A*** and ***B*** correspond to two independent emitting species, each influenced by nuclear dynamics.

The processes which are most likely connected with the respective spectral features are summarized in Fig. 4B. Figure 4B(I) shows the (local) Auger decay upon core-level ionization of ${{\rm{NH}}}_{4}^{+}$ (aq), and Fig. 4B(II.1,II.2) present the Auger and the ICD autoionization channels of the emerging transient structures. The increase of the electron kinetic energy when passing from structure I to structure IV corresponds to gradual delocalization of the charge corresponding to a lower Coulombic repulsion in the respective species. We discuss below the rationale behind this assignment which cannot be established purely on experimental grounds. In the previous studies of neutral molecules, the autoionization electron-signal of *2* *h* aqueous-phase states was determined from a comparison with the respective *2* *h* gas-phase states. The liquid-phase spectrum can hence be constructed from an energy-shifted (due to long-range polarization of the aqueous solution) and broadened (due to the existence of hydration configurations) gas-phase spectrum^5, 12, 15, 16^. For ${{\rm{NH}}}_{4}^{+}$, however, the gas-phase Auger spectrum is not known, and cannot be accessed in the present experiment as the ion is not present in the vapor. Therefore, we must rely on theory in estimating the highest *2* *h* state energy of ${{\rm{NH}}}_{4}^{+}$ (gas).

To obtain a reliable estimate for the *2* *h* Auger peak energies for gas-phase ${{\rm{NH}}}_{4}^{+}$, we performed high-level calculations of the gas-phase energies using the MOM-CCSD(T)/cc-pCVTZ method. We then estimated the solvent energy-shift within a polarizable continuum model (for more details see Methods). The computational protocol was tested for well-studied water and ammonia molecules which are of comparable size to the system studied here. Table 1 compares the calculated and the experimentally measured Auger peak energies. Gas-phase values are consistent within 0.1 eV accuracy. The agreement is slightly less accurate for the liquid phase, but the error is still within 0.5 eV. The estimated value of the highest-energy Auger electrons from gas-phase ${{\rm{NH}}}_{4}^{+}$ is 349.8 eV KE, which is much lower than ammonia (370 eV KE) due to the higher Coulomb repulsion in the intermediate ${{\rm{NH}}}_{4}^{2+}$ ion. The calculated solvent-energy shift for ${{\rm{NH}}}_{4}^{+}$ is approximately 10 eV, which is much larger compared to NH_3_ and H_2_O (~4.5 eV). Hence the Auger peak energy of the *2* *h* state of ${{\rm{NH}}}_{4}^{+}$ (aq) has to be assigned to 361.9 eV KE, which is only 0.6 eV below the center of peak ***A*** (see Fig. 4). In our calculations, we assumed ground-state geometry of the system, thus ignoring the dynamics on the core-ionized state. The importance of the nuclear dynamics for the local peak ***A*** is evidenced by an overall isotope shift of approximately 600 meV indicated in Fig. 4A by small dashed lines. Our MOM calculations show that even gas-phase ammonium cation expands upon core ionization. For the gas-phase molecule the *N*–*H* bonds are elongated by 0.1 Å which leads to a 600 meV shift to higher kinetic energies.

**Table 1:** Calculated and experimental energies of the leading (local) Auger peak for H_2_O, NH_3_ and ${{\rm{NH}}}_{4}^{+}$ in the gas phase and in aqueous solution.

	*E*_Auger_ (gas) [eV]		*E*_Auger_ (aq) [eV]	
	Experiment	Theory	Experiment	Theory
H_2_O	498.6	498.7	503.1	503.6
NH_3_	370.0	369.9	374.0	374.4
${{\rm{NH}}}_{4}^{+}$	not available	349.8	(362.5)	361.9

The calculated gas-phase values were obtained using the MOM-CCSD(T) method with cc-pCVTZ basis set for heavy atoms and cc-pVTZ basis set for hydrogen atoms. The solvent shift was calculated within a polarizable continuum model as described in the Methods, and has been used to determine the theoretical Auger energies for aqueous phase. The molecular geometries were optimized at the MP2/cc-pVTZ level. Experimental values for H_2_O and NH_3_ are taken from refs 5 and 16, respectively. The value for ${{\rm{NH}}}_{4}^{+}$ (aq) is from Fig. 4A (peak ***A***), and the bracket indicates that the peak assignment is assisted by computations; see text for details.

What remains to be discussed is the origin of peak ***B*** (Fig. 4A), and how the accelerated PTM-CS processes relate to it. Previously studied PTM-CS processes for neutral molecules showed only broadening of the main peak (with a small shoulder and no appearance of a second peak)^12^ which is expected due to the exponential decay of the core-ionized population. At *t* = 0 after the ionization event, the only possible structure, with 100% contribution to the autoionization signal, is ${({{\rm{NH}}}_{4}^{2+})}^{\ast }\cdots {{\rm{H}}}_{2}{\rm{O}}$, assigned to peak ***A***. This peak broadens as the $[{({{\rm{NH}}}_{3}^{+})}^{\ast }\cdots {{\rm{H}}}^{+}\cdots {{\rm{H}}}_{2}{\rm{O}}]$ transient species evolve. The population of these transients exponentially decreases for structures with increasing ${({{\rm{NH}}}_{3}^{+})}^{\ast }\cdots {{\rm{H}}}^{+}$ distance as they are formed later in time and autoionization continuously drains the population. The emergence of a new peak ***B*** hence indicates that a new relaxation channel opens within *t* < 6.4 fs, leading to a sudden increase of electron emission near 373 eV KE. Pronounced signal on the high-kinetic energy side of peak ***B*** identifies PTM-CS processes. We thus identify peak ***B*** as the Auger peak of core-ionized ammonia, ${({{\rm{NH}}}_{3}^{+})}^{\ast }$, formed upon a complete proton transfer following the core ionization of ${{\rm{NH}}}_{4}^{+}$ (aq). Qualitatively, this is corroborated by the fact that peak ***B*** almost quantitatively coincides with the spectral positions of the reported *2* *h* (~373 eV, dark-green shade in Fig. 4A) and *1* *h1* *h* (374–387 eV, light-green shades) states of NH_3_ (aq) of ref. 16, resulting from local Auger decay, and PTM-Auger decay and PTM-ICD (with incomplete proton-transfer though)^16^, respectively. We have then to re-rewrite the above Equation (6), and obtain:

7 $$\begin{array}{cc}{\rm{First}}\,({\rm{complete}})\,{\rm{proton}}\,\mathrm{transfer}: & {({{\rm{NH}}}_{4}^{2+})}^{\ast }\cdots {{\rm{H}}}_{2}{\rm{O}}\to {({{\rm{NH}}}_{3}^{+})}^{\ast }+{{\rm{H}}}_{3}{{\rm{O}}}^{+}\end{array},$$

8 $$\begin{array}{cc}{\rm{Second}}\,({\rm{incomplete}})\,{\rm{proton}}\,\mathrm{transfer}: & {({{\rm{NH}}}_{3}^{+})}^{\ast }\cdots {{\rm{H}}}_{2}{\rm{O}}\to [{{(\mathrm{NH}}_{2})}^{\ast }\cdots {{\rm{H}}}^{+}\cdots {{\rm{H}}}_{2}{\rm{O}}]\end{array}.$$

The first proton transfer leads to the formation of core-ionized ammonia ${({{\rm{NH}}}_{3}^{+})}^{\ast }$ and hydronium cation (H_3_O^+^); subsequent local Auger decay (corresponding to peak ***B***) is illustrated in Fig. 4B(III). Autoionization processes of $[{{\rm{NH}}}_{2}^{\ast }\cdots {{\rm{H}}}^{+}\cdots {{\rm{H}}}_{2}{{\rm{O}}}^{+}]$ transient species, formed in an incomplete proton transfer, are depicted in Fig. 4B(IV.1) for Auger decay, and in Fig. 4B(IV.2) for ICD; these emissions are most likely responsible for the signal highlighted by a light-green shade. The only difference with respect to neat NH_3_ (aq) solution is the presence of hydronium cations which may affect the *1* *h1* *h* state energies. But this effect is small as inferred from the almost identical peak ***B*** position. Note that the population of the ${({{\rm{NH}}}_{3}^{+})}^{\ast }$ accumulates as the first proton transfer is almost complete. The proton is then stopped, does not evolve further and the fast electrons are produced for relatively long time interval. The existence of the well separated peak ***B*** thus confirms the full proton transfer.

## Conclusion

We have measured Auger/autoionization-electron spectra from aqueous NH_4_Cl/ND_4_Cl solution. The data are interpreted with *ab initio* calculations and molecular dynamics simulations both in the ground and core-ionized states. The experimental N 1s Auger spectra from ${{\rm{NH}}}_{4}^{+}$ (aq) exhibit a strong local Auger-electron peak, but non-local autoionization (implying two-hole final states with separated charges) contributions to the spectra are of comparable magnitude.

Based on our theoretical analysis, we conclude that the charge separation is dominated by an ultrafast proton transfer on the core-ionized potential energy surface. Upon the core ionization of ${{\rm{NH}}}_{4}^{+}$ (aq), the proton is fully transferred within the very short nitrogen 1s Auger lifetime (6.4 fs). This is the most pronounced charge separation within core-hole lifetime reported to date and can be contrasted with previously studied systems where only partial proton transfer was observed^5, 15, 16^. Hydrated ammonium cation is also the first example in which a double-proton (one complete and one partial) transfer is observed in substantial amount.

Experimentally, the double-proton transfer is reflected by an unique double-peak autoionization spectrum which exhibits large variations in the signal intensities due to the non-local *1* *h1* *h* final states when comparing the N 1s autoionization spectra from ${{\rm{NH}}}_{4}^{+}$ and ${{\rm{ND}}}_{4}^{+}$ aqueous solutions. The first peak (***A***) near 362.5 eV kinetic energy corresponds to the local Auger decay, and the large signal tail on the high-kinetic energy side associated with ${{\rm{NH}}}_{4}^{+}/{{\rm{ND}}}_{4}^{+}$ (aq) arises from transient structures in which the proton is shared with a neighboring water molecule; this corresponds to a time instant where the first proton transfer is not yet complete. The second peak (***B***) near 373 eV is found to almost perfectly coincide with the N 1s autoionization spectrum from NH_3_ (aq). This is a strong evidence of the predicted processes. Specifically, the emergence of the electron signal at 373 eV signalizes a second relaxation channel which only opens when the first proton transfer is finished. Once the first proton transfer is completed and a new chemical bond to form H_3_O^+^ (aq) is made, the remaining core-excited species is ${({{\rm{NH}}}_{3}^{+})}^{\ast }$, i.e. the same species occurring for core-ionized NH_3_ (aq). In other words, the fact that in addition to the *2* *h* and *1* *h1* *h* of ${{\rm{NH}}}_{4}^{+}$ (aq) we also measure the respective local and PTM-CS states of NH_3_ (aq) is a direct experimental confirmation of the ultrafast chemical reaction.

The study of the PTM-CS phenomenon in this work confirms the conclusion drawn from our previous studies that the effect is controlled by the strength of hydrogen bonding. Although the interpretation of the present experimental data is rather non-trivial, the effects reported may lay the groundwork for a new liquid-state spectroscopy. One particular spectroscopy aspect would be the application for studying ion pairing. The non-local charge separation processes should be in fact sensitive to the solution-structure changes induced by added electrolyte. However, the expected effect would be comparatively small as one detects small differences between differential spectra. Ammonium cation seems a very suitable test case for probing the local environment because of the effective charge separation, and ion pairing in ammonium chloride is well characterized^54^. In future studies we will investigate the PTM signal from solutions with various added electrolytes, including multiply charged anions. One particularly appealing system with respect to the double-proton transfer is the oxonium cation, H_3_O^+^. This system is also very interesting in the context of probing the different forms of H^+^ (aq), i.e., Zundel $({{\rm{H}}}_{5}{{\rm{O}}}_{2}^{+})$ and Eigen $({{\rm{H}}}_{9}{{\rm{O}}}_{4}^{+})$ ions, involved in the proton transport mechanism in acidic solutions^55^. Experimentally, this is very challenging because the solute autoionization signal will strongly overlap with the large signal from water.

Investigating the Auger spectra of the ammonium cation (aq) represents a necessary step for exploration of the non-local processes in more complex molecules such as amino acids, in their different protonation states. Also, the role of the charge separation processes in radiation chemistry needs to be further explored; an important issue being the fate of the primary species formed upon the PTM-CS. Answering this question seems, however, beyond the capabilities of Auger spectroscopy. Here, pump-probe techniques^56–58^ using femtosecond X-ray pulses are expected to come into play in future studies and this will also enable actual time-resolved investigations of the dynamical processes in liquid phase. At the moment, the most tangible option though, is to address the issue with theoretical methods.

## Electronic supplementary material

Supplementary info
