# Supplementary material for: Aqueous Solution Chemistry of Ammonium Cation in the Auger Time Window
Source: Sci Rep. 2017 Apr 7;7:756. doi: 10.1038/s41598-017-00756-x (PMC5429669; doi:10.1038/s41598-017-00756-x)
Supplement: Supplementary file 1 — Supplementary info [file 41598_2017_756_MOESM1_ESM.pdf]

Supplementary Information for Article:

Aqueous Solution Chemistry of Ammonium Cation in the Auger Time Window

Daniel Hollas,[1] Marvin N. Pohl,[2,3] Robert Seidel,[2] Emad F. Aziz,[2,3] Petr Slavíček,[1,4,]* and Bernd Winter[2,#,]*

[1] *Department of Physical Chemistry, University of Chemistry and Technology, Prague, Technická 5, 16628 Prague, Czech Republic*

[2] *Helmholtz-Zentrum Berlin für Materialien und Energie, Methods for Material Development, Albert-Einstein-Straße 15, D-12489 Berlin, Germany*

[3] *Department of Physics, Freie Universität Berlin, Arnimallee 14, D-14159 Berlin, Germany*

[4] *J. Heyrovský Institute of Physical Chemistry, Dolejškova 3, 18223 Prague 8, Czech Republic*

[#] *Present address: Fritz-Haber-Institut der Max-Planck-Gesellschaft, Faradayweg 4-6, D-14195 Berlin, Germany*

*Corresponding authors: petr.slavicek@vscht.cz, winter@fhi-berlin.mpg.de

Convergence of electronic structure

It is critical to correctly describe the dynamics of core-ionized ammonium cation. Since our aim is to model the aqueous solution, the method needs to be at the same time sufficiently fast. We have thus used the B3LYP functional. Here we benchmark the approach against calculations obtained at the MP2 level. The comparison is shown in Figure SI1 which compares the single and double-proton transfer for $NH_4^+(H_2O)_3$ clusters upon core ionization. The simulation protocol is described in the Methods section. It is clear that the differences between the MP2 and B3LYP dynamics are rather minor and main qualitative features are preserved, i.e. we see one full proton transfer and one half proton transfer after 7 fs following N 1s ionization.

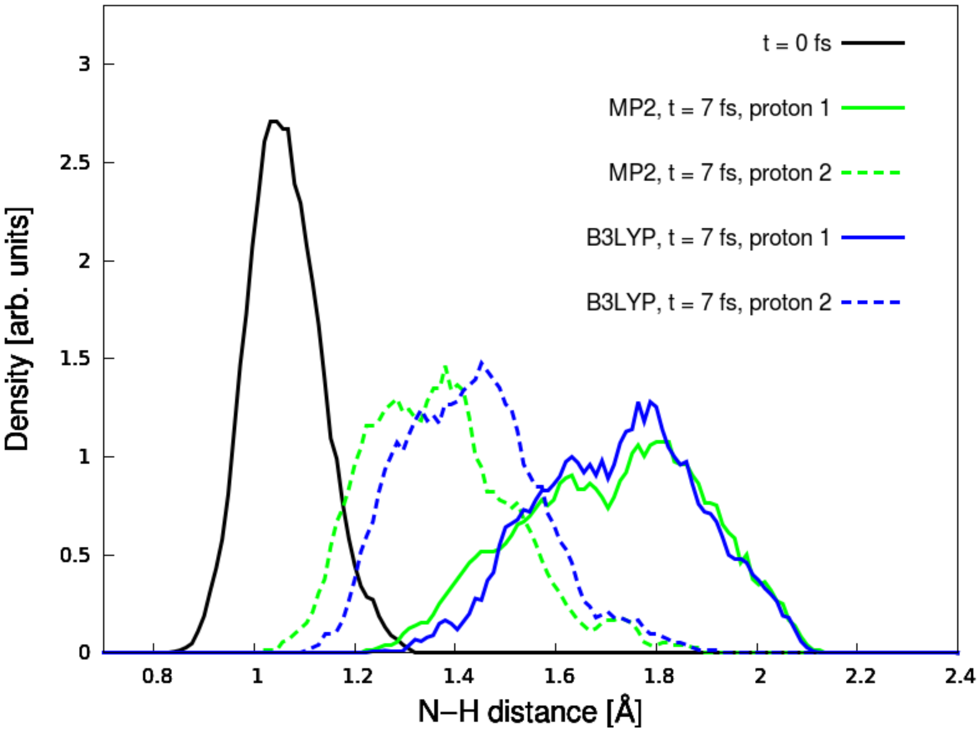

FIGURE SI1: The comparison of electronic structure methods for the description of proton transfer in core-ionized $NH_4^+(H_2O)_3$ clusters. The simulation protocol is described in detail in the Methods section. All simulations utilized the cc-pCVDZ basis set.

Cluster-size dependence of the proton transfer

In simulations of the core-ionized system, we model liquid solution using finite size clusters (although the initial structures were obtained from ground state liquid phase simulations using periodic boundary conditions). It is thus important to ask whether the presented results are robust with respect to the size of the clusters.

Figure SI3 shows 1D potential energy scans of the core-ionized state along the proton-transfer coordinate. The energetics of the proton transfer is rather sensitive to the number of water molecules in the first solvation shell. The energy gain due to the proton transfer gets smaller with increasing number of solvating molecules. However, even in the limit of liquid phase (modelled by Polarizable Continuum model), the proton transfer is still a barrierless and energetically allowed process. Note that this limiting case does not represent a realistic situation as fully equilibrated solvent is considered in this case. This will surely not be the case within the femtosecond duration of the process. The present case thus represents the (artificially) most unfavorable arrangement for the proton transfer which however still takes place.

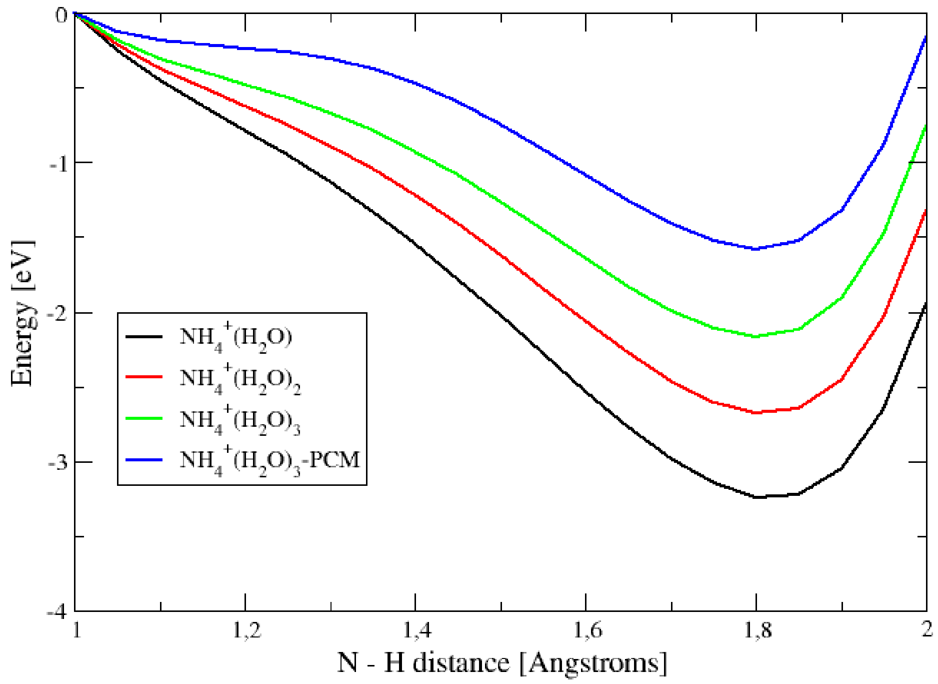

FIGURE SI2: Energy of the N 1s core ionized state as a function of proton-transfer coordinate for different cluster sizes. The curves were calculated at the MP2/cc-pCVDZ level of theory. The geometry of the $NH_4^+(H_2O)_3$ cluster was optimized at the MP2/cc-pVTZ level with counter poise correction. The geometries of smaller clusters were generated from this cluster by removing one or two water molecules, respectively.

The most conclusive evidence of the convergence of the simulations with the number of solvating water molecules comes from the comparison of dynamical results for clusters of different sizes. Figure SI4 shows the extent of single and double-proton transfer upon the N 1s core ionization in $NH_4^+(H_2O)_3$ and $NH_4^+(H_2O)_{20}$ clusters using the B3LYP/cc-pCVDZ method. The differences are rather small. It is in fact a bit surprising that the differences are so small given the large role of the solvation for the energetics of this process (as illustrated in Figure SI2). However, one should keep in mind that it is in fact the initial velocities that are most important for the short-time dynamics (providing the process is barrierless).

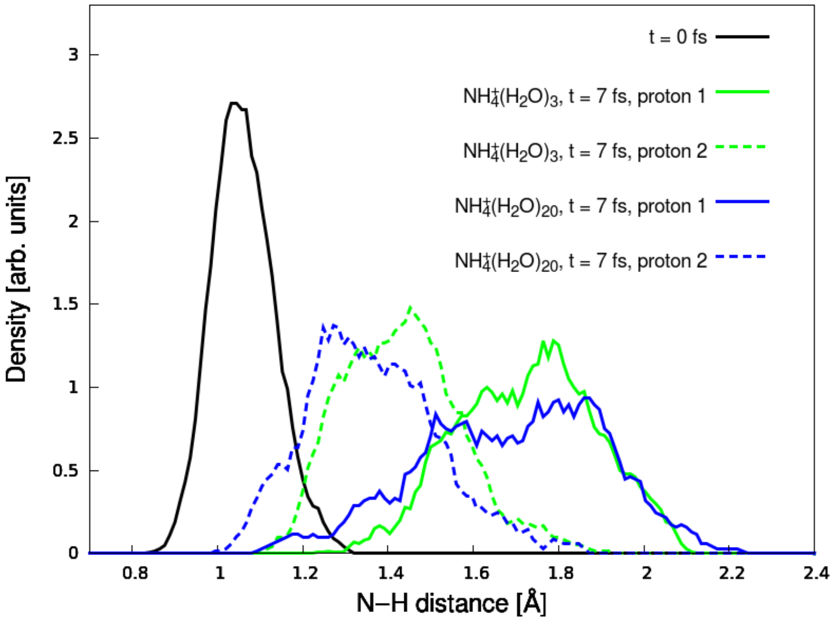

FIGURE SI3: Comparison of the single and double-proton transfer following the N 1s core ionization in $NH_4^+(H_2O)_3$ (green lines) and $NH_4^+(H_2O)_3$ (blue lines) clusters using the B3LYP/cc-pCVDZ method.

Cartesian coordinates of $NH_4^+(H_2O)_3$ cluster

The following geometry of $NH_4^+(H_2O)_3$ cluster was optimized at the MP2/cc-pVTZ level. This geometry was used for potential energy surface scans presented in Fig. 2 and Fig. SI2.

N	0.000142	-0.000122	0.705097
H	0.000129	-0.000130	1.719931
H	0.978030	-0.031718	0.367511
H	-0.461443	0.862562	0.367574
H	-0.516214	-0.831198	0.367548
O	-1.264558	2.301652	-0.226218
H	-0.939705	2.903906	-0.901562
H	-2.050352	2.729457	0.125780
O	-1.361407	-2.245725	-0.226129
H	-2.046100	-2.265237	-0.900769
H	-1.337173	-3.140798	0.124074
O	2.625743	-0.055845	-0.226194
H	2.983642	-0.634083	-0.905810
H	3.389968	0.407438	0.128379
